# Long-term outcome of arthroscopic debridement of massive irreparable rotator cuff tears

**DOI:** 10.1371/journal.pone.0241277

**Published:** 2020-11-12

**Authors:** Tim Vogler, Dimosthenis Andreou, Georg Gosheger, Nico Kurpiers, Clara Velmans, Yacine Ameziane, Kristian Schneider, Carolin Rickert, Dennis Liem, Dominik Schorn

**Affiliations:** 1 Department of General Orthopedics and Tumor Orthopedics, Münster University Hospital, Münster, Germany; 2 Institute of Sports Science, University of Hildesheim, Hildesheim, Germany; Paracelsus Medizinische Privatuniversitat - Nurnberg, GERMANY

## Abstract

**Objectives:**

To evaluate the clinical and radiographic outcome of low-demand patients with massive rotator cuff tears undergoing arthroscopic debridement in mid- and long-term follow-up, as well as the rate of conversion to reverse shoulder arthroplasty.

**Methods:**

We performed a retrospective analysis of 19 patients with a mean age at surgery of 68 years (range, 55–80 years) from a previously described consecutive cohort and after a mean follow up of 47 month (FU1) and 145 month (FU2). The functional outcome was evaluated with the VAS score, the American Shoulder and Elbow Surgeons (ASES) score, and the age- and gender-adjusted Constant (aCS) score. The radiographic outcome was classified according to the Hamada classification. Non-parametric analyses were carried out with the Mann-Whitney U for independent samples and the Wilcoxon signed-rank test for related samples.

**Results:**

Five patients (26%) developed symptomatic cuff tear arthropathy and underwent reverse shoulder arthroplasty after a mean time of 63 months (range, 45–97 months). These patients were excluded from further analyses. The mean VAS score of the remaining 14 patients at FU1 was significantly lower compared to preoperatively (P = .041), while there were no significant differences between the VAS score at FU1 and FU2 (P = 1.0). The ASES score of the affected shoulder at FU1 was significantly higher compared to prior to surgery (P = .028), while there were no significant differences between the scores of the affected shoulder between FU1 and FU2 (P = .878). While the ASES score of the contralateral shoulder at FU1 was significantly higher than the score of the affected shoulder (P = .038), there were no significant differences in the ASES scores of the affected and the healthy shoulder at FU2 (P = .575). The evaluation of the aCS produced similar results. A progression of the Hamada grade was documented in 6 patients.

**Conclusions:**

Arthroscopic debridement is a safe and valid option for low-demand middle-age or elderly patients with symptomatic massive rotator cuff tears, leading to a significant pain relief and significantly improved functional outcome at mid- and long-term follow up. However, about a quarter of the patients in our cohort had to undergo reverse shoulder arthroplasty due to symptomatic cuff tear arthropathy. Furthermore, some of the remaining patients continued to undergo radiographic progression. This might be due to the natural history of their disease and/or the surgical procedure, and the clinical relevance of this finding should be evaluated in further studies.

## Introduction

Rotator cuff tears are a frequent cause of pain and impairment of shoulder function [1, 2]. Their prevalence is estimated at 24% in patients between 40 to 60 years of age and increases up to 54% in patients older than 60 years [3–5]. 10–40% are reported to be massive tears, a term used to describe a tear of a diameter of more than 5 cm [6] or a tear of two or more tendons [7].

Conservative measures such as physical therapy, oral pain medication, or subacromial infiltration are first-line treatment options for patients with low physical demands [2, 8]. If non-operative treatment fails, there are a number of different surgical techniques to improve pain and shoulder function. The choice of the best surgical treatment option is influenced by tissue quality, concomitant osteoarthritis and patient-specific factors, such as age and activity level [9, 10]. The repair of a torn rotator cuff is the favored treatment option and leads to good to excellent clinical results in arthroscopic [11–15] and open procedures [6, 16, 17].

If complete repair cannot be achieved partial repair of the rotator cuff is known to be a reasonable treatment alternative by restoring the shoulder’s force couple and its cable system of force transmission, leading to a biomechanically intact and functional rotator cuff tear [18–20]. Burkhart et al. developed to that end a side-to-side repair–the so-called margin convergence technique–for large longitudinal rotator cuff tears, which has been shown to reduce the strain on the rotator cuff by up to 75% [21, 22].

If complete or partial repair of the rotator cuff tear is not possible, an absorbable subacromial spacer can be implanted arthroscopically to avoid cranial migration of the humeral head [23]. Otherwise more invasive measures like tendon transfers or reverse shoulder arthroplasty can be an option. A tendon transfer can be an option for young patients with irreparable rotator cuff tears [24, 25], while reverse shoulder arthroplasty is an ideal treatment option for patients with a combination of an irreparable cuff tear and osteoarthritis [26]. All of these procedures have been shown to improve shoulder function and achieve pain relief [23–26].

Another treatment option for patients with massive rotator cuff tears and low functional demands is arthroscopic debridement with or without tenotomy of the long head of the biceps, which is best suited for older patients with pain as their chief complaint [27, 28]. Major advantages of this technique are a shorter duration of surgery, a lower risk of perioperative complications, as well as faster rehabilitation [10]. Liem et al. previously reported the mid-term clinical results of arthroscopic debridement and tenotomy of the long head of the biceps of a series of 31 consecutive patients with an irreparable rotator cuff tear after a mean follow-up of 47 months, and demonstrated a significant reduction of pain and a significant improvement of shoulder function [10]. However, to our knowledge, there are no studies reporting on the long-term outcome of patients treated by this procedure. We therefore performed this study, in order to evaluate whether the good clinical and radiographic mid-term results are sustained in long-term follow-up, as well as to determine the rate of conversion to reverse shoulder arthroplasty. We hypothesized that the functional outcome in long-term follow-up would be comparable to the mid-term results.

## Materials and methods

### Ethics statement

The study was approved by the local ethics committee (Ethikkommission der Ärztekammer Westfallen-Lippe und der Westfälischen Wilhelms-Universität), approval number: 2015-709-f-S. All patients provided written informed consent prior to study enrollment.

### Patient population

For this retrospective analysis we attempted to contact and reexamine all 31 consecutive patients included in the previously mentioned study by Liem et al. [10] which was performed at our institution. These patients had received arthroscopic debridement and biceps tenotomy between 2003 and 2004 due to an irreparable rotator cuff tear [10]. During this period we performed 126 rotator cuff repairs. Eight of the 31 patients (26%) had died between the first (FU1) and the second (FU2) follow-up examination and 4 patients (13%) were lost to follow-up, leaving 19 patients (61%) as the subject of this study. All patients provided written informed consent prior to study enrollment. The study was approved by the local ethics committee (reference number 2015-709-f-S) and performed in accordance with the Declaration of Helsinki.

Patient demographics are summarized in Table 1. All patients had a massive rotator cuff tear with persistent complaints despite sole conservative treatment with intensive mobilization attempts under physiotherapy guidance over a period of at least 3 months. The persisting symptoms were the reason for surgical treatment. Tears were classified as massive if the supraspinatus tendon was retracted behind the glenoid or involved more than 1 tendon [10]. Due to the size of the rupture, no rotator cuff repair could be performed in our cohort. All patients were able to elevate their shoulder to at least 90 degrees, while lag signs for external and internal rotation and the drop-arm sign were all negative [10]. All patient had low functional demands of their shoulder and were inclined to accept functional deficits, provided that the surgical treatment would provide pain-relief.

**Table 1:** Demographic data and intraoperative findings.

Age at surgery (in years)	68 (range, 55–80)
Gender	
male	9 (47%)
female	10 (53%)
Involved tendons	
supraspinatus	19 (100%)
infraspinatus	15 (79%)
subscapularis	9 (47%)
Biceps	
tenotomy	17 (89%)
rupture	2 (11%)
VAS score prior to surgery	7.4 (range, 7–8)

Patients with shoulder infections, frozen shoulders or prior shoulder surgeries were not included in this study. Radiographic exclusion criteria were grade- II or -III osteoarthritis, according to the Samilson-Prieto classification [29] and an acromiohumeral distance of less than 5 mm before surgery.

### Surgical technique

For surgery the patient was placed in the beach chair position. A diagnostic arthroscopy was first performed, during which the diagnosis of an irreparable rotator cuff tear was confirmed. The state of the biceps tendon was subsequently evaluated. Seventeen patients had signs of synovitis, partial tearing or displacement out of the bicipital groove and underwent biceps tenotomy. The biceps tendon was already ruptured in 2 patients, in which cases the remaining tendon parts were removed from the superior labrum [10]. Arthroscopic debridement of the avascular tendon edges, combined with a bursectomy and partial synovectomy, was then performed in all patients. Neither an acromioplasty nor a resection of the coracoacromial ligament was performed in any patient, in order to maintain the coracoacromial arch.

After surgery all patients were allowed immediate active and passive mobilization of the shoulder as tolerated, while no patient was treated with an orthosis.

### Clinical and radiographic evaluation

The functional outcome was evaluated with the VAS score, the American Shoulder and Elbow Surgeons (ASES) score and the age- and gender-adjusted Constant score (aCS). All scores were evaluated at two follow-up examinations. The first follow-up-examination (FU1) was performed at an average of 47 months (range, 28–62 months) after surgery, while the second follow-up-examination (FU2) of the patients who had not undergone reverse shoulder arthroplasty due to symptomatic cuff tear arthropathy took place at a mean of 145 months (range, 122–170 months) after surgical treatment. For statistical analysis we also used the ASES score prior to operation, which was documented in the patients’ files.

The radiographic outcome was evaluated by two experienced orthopedic surgeons, according to the Hamada classification on standard anteroposterior shoulder radiographs prior to surgical treatment and at FU2. The Hamada classification is commonly used to categorize the radiographic alterations associated with massive rotator cuff tears, based on the decrease in the acromiohumeral intervall, as well as the presence of acromial acetabulization and glenohumeral arthritis [30].

### Statistical analysis

Continuous variables were checked for normality using the Shapiro-Wilk test. Non-parametric analyses were carried out with the Wilcoxon signed-rank test for related samples. The correlation between the decrease in acromiohumeral distance at FU2 and aCS was investigated using Pearson’s r. Statistical analyses were performed with the IBM SPSS Statistics software version 21.0 (IBM Corp., Armonk, NY). All P values are two-sided; a P value ≤.05 was considered significant.

## Results

### Treatment failures

A total of 5 patients (26%) developed symptomatic cuff tear arthropathy and underwent reverse shoulder arthroplasty after a mean time of 63 months (range, 45–97 months) after arthroscopic debridement. These patients were considered as treatment failures and were excluded from the analyses of the clinical, functional and radiographic outcome, which were performed on the remaining 14 patients who underwent no further surgical procedures after the initial arthroscopic debridement.

### Long-term clinical and functional outcome of arthroscopic debridement only

No procedure-related complications were reported. The mean VAS score of the affected shoulder at FU1 amounted to 3.3 (range, 0–9; median, 4), and was significantly lower compared to the VAS score prior to surgery (mean, 7.4; range, 7–8; median, 7; P = .041). There were no significant differences between the VAS score at FU1 and FU2 (mean, 3.4; range, 0–9; median, 3; P = 1.0).

The ASES score of the affected shoulder at FU1 was significantly higher compared to the ASES score prior to surgery, while there were no significant differences between the scores of the affected shoulder between FU1 and FU2 (Table 2). The ASES score of the contralateral shoulder was not documented preoperatively. While the ASES score of the contralateral shoulder at FU1 was significantly higher than the score of the affected shoulder, there were no significant differences in the ASES scores of the affected and the healthy shoulder at FU2, owing to a significant decrease of the score of the contralateral shoulder between FU1 and FU2 (Table 2).

**Table 2:** Mean ASES Score (in brackets range and median) prior to surgery, at FU1 and at FU2, as well as P values (Wilcoxon signed-rank test).

	prior to surgery	FU1	FU2	P value (FU1 vs. prior to surgery)	P value (FU2 vs. FU1)
**affected shoulder**	36 (20–83; 27)	66 (43–95; 59)	66 (12–100; 68)	.028	.878
**contralateral shoulder**	-	82 (67–88; 88)	70 (12–100; 68)	-	.05
**P value (affected vs. contralateral)**	-	.038	.575	-	-

The evaluation of the aCS largely produced similar results. While the score of the contralateral shoulder was significantly higher compared to the score of the affected shoulder at FU1, there were no significant differences at FU2 owing to a significant decrease of the score of the healthy shoulder between FU1 and FU2 (Table 3).

**Table 3:** Mean age- and gender-adjusted Constant Score (in brackets range and median) at FU1 and FU2, as well as P values (Wilcoxon signed-rank test).

	FU1	FU2	P value (FU2 vs. FU1)
**affected shoulder**	79% (51–114; 76%)	63% (25–99; 65%)	.074
**contralateral shoulder**	97% (34–116; 107%)	68% (27–99; 75%)	.008
**P value (affected vs. contralateral)**	.038	.209	-

In order to assess the reasons for the differences in functional outcome between the affected and the contralateral shoulder at FU1 and for the decline in outcome of the contralateral shoulder between FU1 and FU2, we examined the differences in the subjective and objective parameters composing the aCS. The affected shoulder scored significantly lower than the healthy shoulder at FU1 in strength and pain, while the lower score in the evaluation of the activities of daily living did not reach statistical significance with the number of patients available for our analysis (Table 4). The affected shoulder only scored significantly lower in range of motion at FU2 compared to FU1, while the contralateral shoulder had significantly lower scores in the evaluation of the activities of daily living, range of motion and strength at FU2 compared to FU1 (Table 5). The pain score at FU2 was lower compared to FU1 but this did not reach statistical significance.

**Table 4:** Comparisons of the mean values of the aCS composing parameters (in brackets range and median) between the affected and the contralateral side at FU1, as well as between the affected and the contralateral side at FU2, with the respective P values (Wilcoxon signed-rank test).

	FU1 (affected side)	FU1 (contralateral side)	P value	FU2 (affected side)	FU2 (contralateral side)	P value
**Pain**	9.8 (4–15; 10)	12.6 (6–15; 15)	.042	10 (2–15; 9)	10 (2–15; 11)	.893
**activity of daily living**	12.4 (8–20; 10)	16.8 (8–20; 20)	.058	11.2 (3–18; 11.5)	11.5 (3–18; 11.5)	.317
**range of motion**	34.9 (22–40; 38)	34.2 (10–40; 38)	1.0	25.9 (10–38; 26)	28.1 (8–38; 30)	.326
**Strength**	7.1 (2–13; 7)	18.9 (0–25; 22)	.011	6 (0–14; 4)	7.9 (0–14; 8.5)	.059

**Table 5:** Comparisons of the mean values of the aCS composing parameters (in brackets range and median) of the affected side between FU1 and FU2, as well as of the mean values of the contralateral side between FU1 and FU2, with the respective P values (Wilcoxon signed-rank test).

	FU1 (affected side)	FU2 (affected side)	P value	FU1 (contralateral side)	FU2 (contralateral side)	P value
**Pain**	9.8 (4–15; 10)	10 (2–15; 9)	.725	12.6 (6–15; 15)	10 (2–15; 11)	.058
**activity of daily living**	12.4 (8–20; 10)	11.2 (3–18; 11.5)	.313	16.8 (8–20; 20)	11.5 (3–18; 11.5)	.007
**range of motion**	34.9 (22–40; 38)	25.9 (10–38; 26)	.013	34.2 (10–40; 38)	28.1 (8–38; 30)	.012
**strength**	7.1 (2–13; 7)	6 (0–14; 4)	1.0	18.9 (0–25; 22)	7.9 (0–14; 8.5)	.015

### Radiographic outcome

Four of the patients were not willing to undergo radiographic examination at FU2. In the remaining 10 patients the acromiohumeral distance significantly decreased from 10 mm (range, 4–15; median, 9 mm) prior to surgery to 6 mm (range, 2–12; median, 5 mm) at FU2 (P = .007). There was a trend towards a negative correlation between the decrease in acromiohumeral distance and aCS (r = -0.578, P = .062).

**Fig 1:**
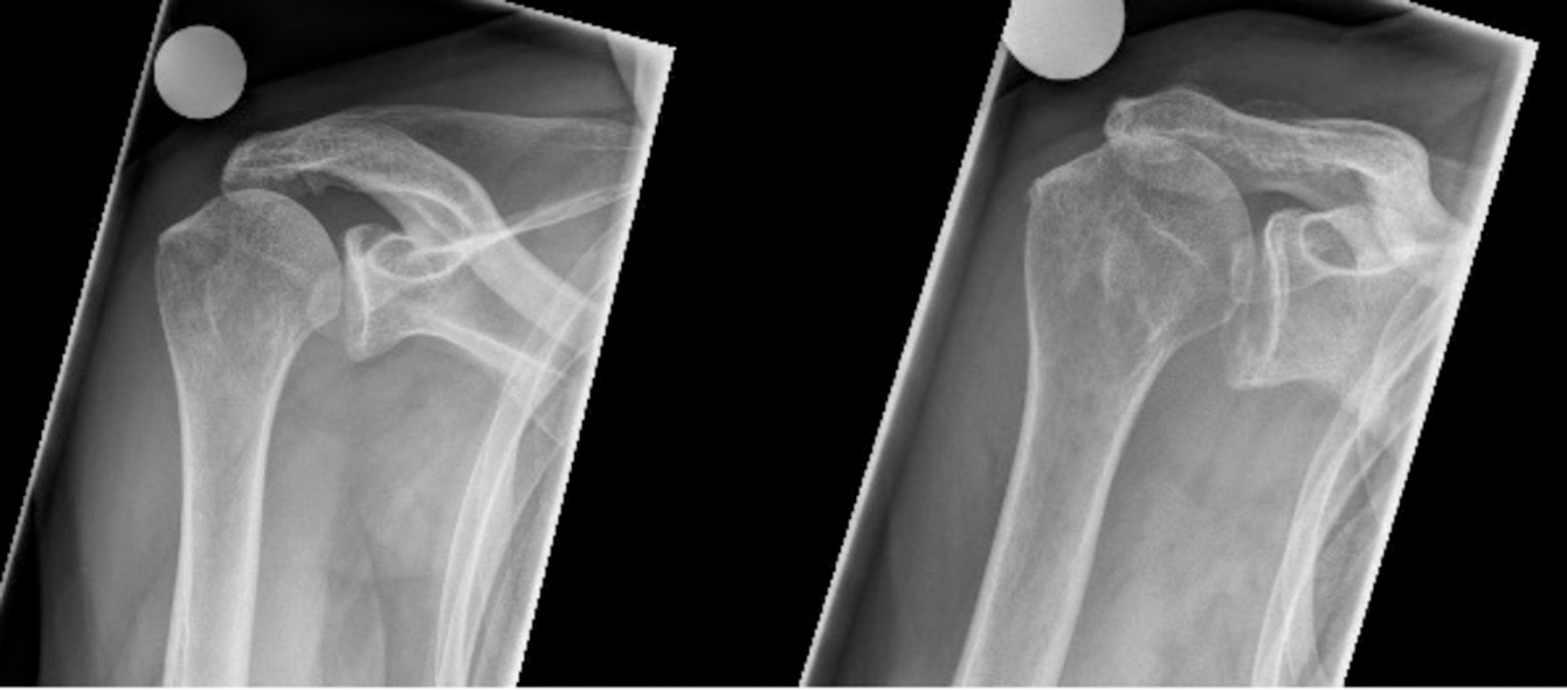
Preoperative (left) radiograph of a 74-year-old male patient with a Hamada grade 2; postoperative (right) radiograph showing a progression of the Hamada grade to 3 after 11 years.

**Fig 2:**
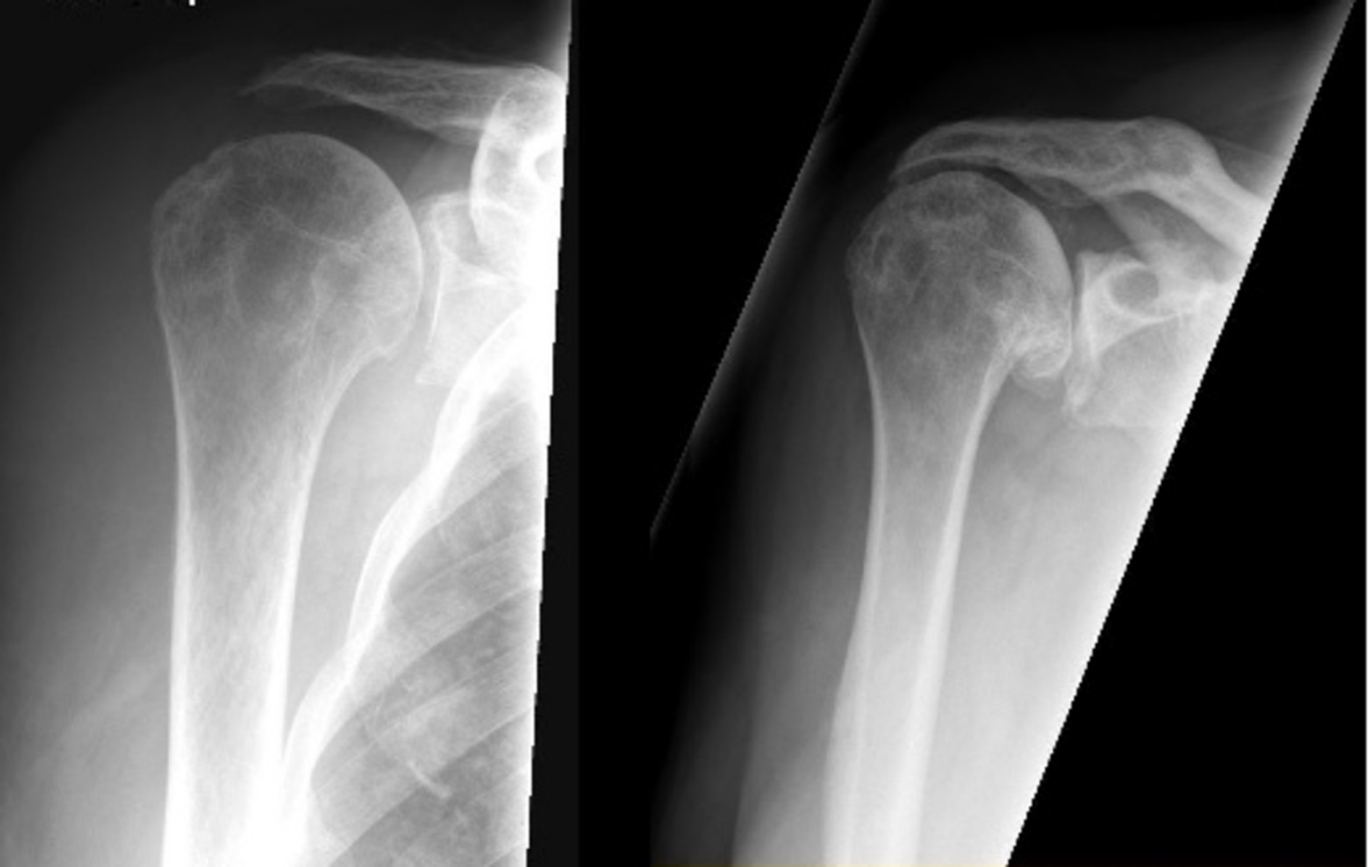
Preoperative (left) radiograph of a 79-year-old female patient with a Hamada grade 2; postoperative (right) radiograph showing a progression of the Hamada grade to 4(B) after 10 years.

Regarding the grade of cuff tear arthropathy, a progression of the Hamada grade was documented in 6 patients. Eight patients had a Hamada grade 1 and two patients a Hamada grade 2 prior to surgery, while at FU2 only three patients had a grade 1, one patient each had a grade 2, 3 (Fig 1) and 5, while two patients each had a grade 4(A) and 4(B) (Fig 2), respectively. However, two of the three patients with the highest aCS at FU2 had a Hamada grade 4(B).

## Discussion

A multitude of surgical techniques have been described for the management of irreparable rotator cuff tears, including debridement [31, 32], partial repair [21, 22], tuberoplasty [24, 33, 34], tendon transfer [24, 35] or prosthetic replacement [36, 37]. Heuberer et al. showed, that reparability of massive rotator cuff tears was influenced by the fatty degeneration of the muscles. In short-term follow up, complete repair showed more favourable improvements than partial repair or arthroscopic debridement [38]. Arthroscopic debridement has shown good mid-term functional results in middle-age or elderly patients with low functional demands suffering primarily from local pain and our study demonstrated that long-term results are equally good.

About a quarter of the patients in our cohort had to undergo reverse shoulder arthroplasty after a mean time of approximately 5 years following arthroscopic debridement. Taking into consideration that arthroscopic debridement was performed in order to avoid endoprosthetic replacement, these patients have to be classified as treatment failures. On the other hand, all of the patients in our cohort had persistent complaints after conservative treatment, and the most suitable surgical options available, taking into consideration their age, were arthroscopic debridement or primary reverse shoulder arthroplasty. Future studies should try to identify possible risk factors at presentation for patients who need conversion to reverse shoulder arthroplasty–something we were unable to do given the retrospective nature of our study and the low number of patients in our cohort.

Our study was also, to our knowledge, the first to research the long-term functional outcome of sole arthroscopic debridement over a period of more than ten years. Pander et al. could prove a high satisfactory shoulder function in elderly patients after 5–10 years in both arthroscopic debridement and debridement combined with biceps tenotomy [39]. Our results show that the good mid-term functional results are sustained in long-term follow-up in most of the patients who did not have to undergo reverse shoulder arthroplasty, with only the range of motion of the affected shoulder showing a significant decline between FU1 and FU2. Furthermore, despite the significantly inferior function of the affected compared to the contralateral shoulder at mid-term follow-up, no significant differences in the aCS and the ASES scores of both shoulders could be detected at long-term follow-up. This discrepancy was owed to a significant reduction in nearly all functional parameters of the healthy shoulder between mid- and long-term follow-up, which might be attributable to the contralateral side also developing a rotator cuff tear over time, as the result of the natural evolution of the rotator cuff with advancing patient age [3–5]. However, our data can neither confirm nor refute this hypothesis. The analysis of the aCS composing parameters of both shoulders at FU2 showed a decreased strength of the affected shoulder compared to the contralateral side without reaching statistical significance, which is likely to be of little relevance to the low-demand patients comprising our cohort.

There was a significant decrease in the acromiohumeral distance from 10mm prior to surgery to 6 mm at long-term follow-up in our patient cohort. We also found a negative correlation between the acromiohumeral distance and the aCS, which, failed to reach statistical significance with the numbers of patients available for this analysis, confirming the prognostic relevance of the acromiohumeral distance. Scheibel et al. [33] have also demonstrated a significant decrease in acromiohumeral distance in a series of 23 patients with massive rotator cuff tears undergoing arthroscopic debridement of the subacromial space and glenohumeral joint combined with an arthroscopic tuberoplasty. Contrary to our analysis, their study included 5 patients with no pathological changes of the biceps tendon, in whom the tendon was preserved. The authors found no changes in the mean acromiohumeral distance of these 5 patients and suggested that the decrease in acromiohumeral distance might be a result of the biceps tendon tenotomy itself, rather than a biological weakening of the remaining rotator cuff [33]. In contrast to that, Klinger et al. found no difference between arthroscopic debridement alone and combined with LHB-tenotomy after 31 month. The results also showed no humeral head migration in both groups [40].

Six of the 10 patients willing to undergo a radiographic examination at FU2 had a progressive cuff tear arthropathy with higher Hamada grades compared to prior to surgery. Interestingly, two of the three patients with the highest aCS at FU2 had a Hamada grade of 4(B), suggesting that the functional outcome after arthroscopic debridement for massive cuff tear might not be related to the cuff tear arthropathy as measured by the Hamada classification.

In recent years, several novel arthroscopic procedures have been established for the treatment of irreparable rotator cuff tears, such as subacromial spacer implantation [23] and superior capsular reconstruction [41]. However, these techniques are associated with a longer duration of surgery and a more complex postoperative treatment, while only short- to mid-term clinical results are available in the literature [23]. The long-term data provided by our analysis can serve as a comparative basis to assess the possible benefits and disadvantages of these newer procedures in terms of functional outcome, taking into consideration procedure-related complications.

Given the low number of patients who are good candidates for this procedure combined with the advanced patient age at surgery, long-term results can be evaluated in only small patient cohorts, which was one of the limitations of our study and makes any comparative statistics limited. Another limitation is the retrospective nature of the study and the lack of a control group. Finally, given the fact that some patients were lost to follow-up, we have to acknowledge a possible selection bias.

In conclusion, arthroscopic debridement combined with biceps tenotomy is a safe and valid option for low-demand middle-age or elderly patients with symptomatic massive rotator cuff tears, leading to a significant pain relief and significantly improved functional outcome at mid- and long-term follow-up. However, about a quarter of the patients in our cohort had to undergo reverse shoulder arthroplasty due to symptomatic cuff tear arthropathy. Furthermore, patients should be informed that at least some of the remaining patients will experience radiographic degeneration over the next ten years, which may be due to the natural history of their rotator cuff tear and/or the surgical procedure. The clinical relevance of this finding should be evaluated in further studies.
